# Antiviral regulator TRIM25 as a prognostic marker of better survival in Merkel cell carcinoma: Association with MCPyV status

**DOI:** 10.1002/ijc.70384

**Published:** 2026-02-14

**Authors:** Klaus W. Fagerstedt, Sami Kilpinen, Johanna Arola, Benjamin Z. Sundqvist, Tom Böhling, Leif C. Andersson, Harri Sihto

**Affiliations:** ^1^ Department of Pathology University of Helsinki and Helsinki University Hospital Helsinki Finland; ^2^ Molecular and Integrative Biosciences Research Programme University of Helsinki Helsinki Finland; ^3^ HUS Diagnostic Centre, Department of Pathology University of Helsinki and Helsinki University Hospital Helsinki Finland

**Keywords:** Merkel cell carcinoma, TRIM25, viral infection

## Abstract

Merkel cell carcinoma (MCC) is a highly aggressive neuroendocrine skin cancer, most often driven by integration of Merkel cell polyomavirus (MCPyV). While anti‐PD‐1/PD‐L1 therapies have improved outcomes, reliable prognostic biomarkers remain limited. Tripartite motif‐containing protein 25 (TRIM25), a critical activator of innate immunity, was evaluated here for its clinical and biological relevance in MCC. We analysed TRIM25 mRNA and protein expression in 102 MCC cases and 9 MCC cell lines, respectively, and assessed associations with MCPyV status, clinicopathological characteristics and patient survival. Differential gene expression analysis was performed to identify pathways associated with *TRIM25* expression in both low and high *TRIM25*‐expressing tumour groups. High TRIM25 expression was significantly associated with MCPyV positivity in both patient tumours (*p* = .004) and cell lines (*p* = .016), and TRIM25 and MCPyV mRNA levels correlated positively in tumours (*r* = 0.264, *p* = .013). Patients with low *TRIM25* expression had a significantly poorer 5‐year disease‐specific survival than those with high expression (53% vs. 78%, *p* = .013). Notably, Cox multivariate analysis confirmed that *TRIM25* serves as an independent prognostic marker, irrespective of MCPyV status. Pathway analysis revealed that low *TRIM25* expression was linked to antigen presentation pathways, while high *TRIM25* expression was associated with cell cycle regulation. Our findings suggest that TRIM25 serves as a valuable prognostic biomarker in MCC. Additionally, TRIM25 may play a pathogenic role in MCPyV‐positive tumours, warranting further investigation to elucidate its mechanistic involvement in MCC tumourigenesis.

## Graphical abstract

What's new?

TRIM25 is known for its role in antiviral immunity and association with poor prognosis in various cancers. This study reveals an association between high *TRIM25* expression and Merkel cell polyomavirus positivity in Merkel cell carcinoma. Moreover, low *TRIM25* expression correlated with poor prognosis, especially in polyomavirus‐negative cases, suggesting its potential as a prognostic marker. Pathway analysis showed that low *TRIM25* expression was linked to antigen presentation pathways, while high *TRIM25* expression was associated with cell cycle regulation. The findings highlight potential functional differences linked to *TRIM25* expression that could influence tumour behaviour and patient outcomes in Merkel cell carcinoma.

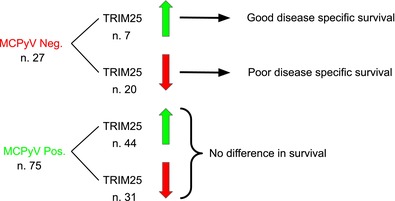

**Abbreviations**
BKV: BK virus
DEG: differentially expressed genes
HBV: Hepatitis B virus
HCC: hepatocellular carcinoma
HPV: human papilloma virus
ICI: immune checkpoint inhibition
IFN: interferon
JCV: JC virus
LOGFC: logarithmic fold change
LT: large T
MAVS: mitochondrial antiviral‐signalling protein
MCC: Merkel cell carcinoma
MCPyV: Merkel cell polyoma virus
MHC‐1: major histocompatibility complex 1
MTA‐1: metastasis‐associated protein 1
NCBI: National Center for Biotechnology Information
PD‐1: programmed cell death protein 1
PD‐L1: programmed cell death protein 1 ligand
RIG‐1: retinoic acid‐inducible gene 1
RNA: ribonucleic acid
SPRY: SPla and Ryanodine receptor
SRA: sequence read archive
TGF‐*ß*
: transforming growth factor beta
TIL: tumour infiltrating lymphocyte
TMB: tumour mutation burden
TP53: tumour protein 53
TRIM25: tripartite motif‐containing protein 25
UV: ultraviolet

## 1 INTRODUCTION

Merkel cell carcinoma (MCC) is a rare neuroendocrine tumour of the skin, first characterised by Cyril Toker in 1972 as ‘trabecular carcinoma of the skin’. MCC is known for its aggressive nature; as noted by Becker et al.,^1^ its mortality rate surpasses that of melanoma, with nearly one‐third of patients presenting with locoregional metastases at the time of diagnosis. MCC predominantly affects individuals aged 75 years and older. Since the early 2000s, the incidence of MCC has ranged from 0.1 to 2.5 cases per 100,000 individuals per year. The highest rates are observed in Australia (3.9/100,000), regions characterised by high UV exposure and a significant proportion of fair‐skinned individuals. A systematic review by Mohsen et al.^2^ indicates that the incidence of MCC has been increasing since the 1990s.^3^ This increase in incidence may be attributed to improved diagnostic techniques, an ageing population, and the use of immunosuppressive medications.

Two primary pathogenic mechanisms have been identified in the development of MCC. The first, common to many skin cancers, is exposure to ultraviolet (UV) light. The second involves genomic integration of the Merkel cell polyomavirus (MCPyV) that is found in approximately 80% of tumours.^2^, ^4^ The MCPyV‐negative tumours have a higher mutation burden than their MCPyV‐positive counterparts.^5^ A high tumour‐infiltrating T‐cell count is associated with a favourable outcome in MCC patients.^6^ Treatment with programmed cell death inhibitor (PD‐1) in patients with advanced MCC has yielded a good response, with a median progression‐free survival of 16.8 months.^7^ Pembrolizumab has been shown to have a better effect on tumours with low mutation burden and in MCPyV‐positive patients with an objective response rate (ORR) of 59% compared with MCPyV‐negative tumours with an ORR of 53%.^8^ The PD‐1 ligand (PD‐L1) targeting avelumab has been shown to have an overall response rate of 33% and most of the longtime survivors had PDL‐1 expressing tumours.^8^

The tripartite motif‐containing protein 25 (*TRIM25*) is a member of a gene family comprising over 70 members. TRIM25 plays a critical role in the regulation of the innate immune response to viral infection through ubiquitination. TRIM25 functions as an E3 ubiquitin ligase, specifically targeting the SPla and Ryanodine receptor (SPRY) domain of RNA sensor RIG‐I (RIG‐I). This interaction facilitates the formation of the Mitochondrial Antiviral Signalling Protein‐RIG‐I (MAVS‐RIG‐I) complex, which promotes the production of type I and III interferons (IFNs).^9^, ^10^, ^11^, ^12^ IFNs then limit the replication and spreading of the virus.^11^, ^13^ However, many virus strains have evolved mechanisms to downregulate the innate immunity by modulating TRIM25 or its associated proteins, which may lead to chronic infection, supporting tumourigenesis.^13^, ^14^ For example, the E6 oncoprotein of human papilloma viruses can form a complex with TRIM25, resulting in increased ubiquitination and degradation of TRIM25, and ultimately downregulation of RIG‐I activation.^13^ In contrast, the small T‐antigens of BKV and JCV polyomaviruses can bind to TRIM25, preventing its binding ability to RNA and induction of RIG‐I ubiquitination.^14^

Elevated *TRIM25* expression has been associated with increased cell migration, invasion, cellular proliferation, and cellular survival in various cancers using, for example, the TGF‐*ß* signalling pathway.^15^, ^16^, ^17^, ^18^ In hepatocellular carcinoma, elevated TRIM25 is also correlated with poorer clinical outcomes.^15^ TRIM25 has been proposed as a potential therapeutic target and biomarker in breast cancer because its gene expression is modulated by oestrogen.^19^ Vise versa high TRIM25 expression is also implicated in cisplatin and oxaliplatin chemoresistance.^20^, ^21^

This study aims to investigate the impact of *TRIM25* expression in MCC, where tumourigenesis is frequently driven by MCPyV. Here we examined the association between TRIM25 mRNA expression and patient survival and clinicopathological variables in 102 MCPyV‐negative and MCPyV‐positive MCCs, and TRIM25 protein expression in nine MCC cell lines. Survival analyses addressing the relationship between MCC and TRIM25 expression have not been previously conducted.

## 2 MATERIALS AND METHODS

### 2.1 Patient material

The retrospective sample series consisted of 102 MCC cases from Finland, diagnosed between 1983 and 2013. Patient data were obtained from the Finnish Cancer Registry and the files of Helsinki University Hospital, and tissue samples from pathology archives, as detailed elsewhere.^22^, ^23^ Presence of MCPyV DNA in tumour tissue was detected using quantitative PCR.^23^ RNA was extracted and sequenced as described in detail previously.^22^ Sequencing was conducted on 120 formalin‐fixed, paraffin‐embedded samples, of which 14 cases did not pass the quality control analysis of the transcriptome data, and two cases were missing MCPyV status, resulting in the inclusion of 102 MCC cases in the study. The median follow‐up time for all patients was 2.98 years (range 13 days to 33 years). Regarding sun exposure categorisation, the head and neck areas were considered sun‐exposed areas, while the limbs and torso were classified as non‐sun‐exposed areas.

### 2.2 Differential expression analysis

The samples were divided into groups with low *TRIM25* and high *TRIM25* expression and differentially expressed genes (DEGs) within, and the groups were analysed as follows: transcriptomes of MCC patients were extracted from the SRA database in NCBI (BioProject PRJNA775071). A design matrix was made by using binary grouping of the variable TRIM25 (low or high) based on its median threshold. A linear model was fit using the limma software package in R.^24^ For model fitting, the logCPM expression matrix was used as input for ImFit to fit linear models for each gene. eBayes was used to compute moderated *t*‐statistics, *p*‐values, and log fold changes (logFC) for the DEGs in *TRIM25* low and *TRIM25* high groups. A volcano plot was generated to visualise DEGs, with the *x*‐axis having Log2 fold change (logFC) and the *y*‐axis having Log10 adjusted *p*‐value. A dashed line indicates an adjusted *p*‐value of .05. Gene symbols of the top 100 genes with the highest absolute logFC values were annotated to the image. Genes were considered differentially expressed if their adjusted *p*‐value (Benjamini–Hochberg corrected) was less than 0.05. Lists of significant DEGs were saved for further interpretation. This was done by analysing the genes' relations with Enrichr^25^, ^26^, ^27^ with Bioplanet 2019 pathways (access date: 12/12/2024).

### 2.3 Viral mRNA load

As TRIM25 is a known component of antiviral innate immune responses, we aimed to investigate whether transcriptionally active MCPyV integrated in the tumour cell genome is associated with TRIM25 expression levels. To address this, we analysed viral mRNA abundance reflecting overall viral transcriptional activity in tumour tissues by using nf‐core/viralintegration pipeline (doi: 10.5281/zenodo.7783480) with specific single‐end sequencing suitable code branch (https://github.com/nf-core/viralintegration/tree/single-end) of version 0.1.1 (10.5281/zenodo.7783480, 10.1038/s41587‐020‐0439‐x). The analysis pipeline aligns RNA sequencing reads against the human genome reference GRCh37 combined with viral genomes provided by the pipeline, identifying viral‐host chimeric transcripts indicative of integrated viral genomes that are transcriptionally active. Further parameter adjustments were done to adapt to QuantSeq read length and adapter trimming. Entire pipeline frozen at the version used in the study can be found from (https://github.com/Rare-Cancers-Research-Group/Porocarcinoma/tree/main/code/viral-integration/viralintegration-local). Pipeline return data only for samples in which a meaningful amount of reads for any viral genomes was detected. Code for downstream data processing is available at (https://github.com/Rare-Cancers-Research-Group/MMC-TRIM25.git). Downstream analysis of viral loads per sample was based on frac_covered value per sample for MCPyV viral genome as returned by the pipeline. Correlation analysis between viral load (log1p transformed) and TRIM25 expression was performed using Pearson's correlation coefficient, reflecting potential biological interplay between viral integration transcriptional activity and host antiviral response mediated by TRIM25.

### 2.4 Cell lines

MCPyV‐positive MCC cell lines WaGa (RRID:CVCL_E998), PeTa (RRID:CVCL_LC73), and MKL‐2 (RRID:CVCL_D027) were kindly provided by Dr. Roland Houben (University Hospital Würzburg, Germany). Virus‐positive cell lines MS‐1 (RRID:CVCL_E995) and MKL‐1 (RRID:CVCL_2600) and virus‐negative cell lines MCC13 (RRID:CVCL_2583), MCC14/2 (RRID:CVCL_2584), and MCC26 (RRID:CVCL_2585) were acquired from the European Collection of Authenticated Cell Cultures (ECACC). Virus‐negative cell line UISO‐MCC‐1 (RRID:CVCL_E996) was kindly provided by Prof. Annamari Ranki (Helsinki University Hospital, Finland). All cell lines were authenticated using short tandem repeat (STR) profiling within the past year in the genotyping lab of the FIMM Technology Centre (Helsinki, Finland). All experiments were performed with mycoplasma‐free cells. Cell lines were cultured in a humidified, 5% CO_2_ atmosphere at 37°C. All cell lines were cultured in Gibco™ RPMI 1640 Medium with 1× Gibco™ Penicillin–Streptomycin‐Glutamine, and heat inactivated 10% Gibco™ fetal bovine serum (Life Technologies, Carlsbad, CA, USA).

### 2.5 Western blotting

The cells were lysed in M‐PER™ Mammalian Protein Extraction Reagent (Cat. No. 78501, Thermo Fisher Scientific Inc., MA, USA) supplemented with HALT™ protease and phosphatase inhibitor cocktails (Cat. No. 78429 and Cat. No. 78420, Thermo Fisher Scientific). Lysates were separated by SDS‐PAGE using Mini‐PROTEAN® TGX™ Precast Gels (Cat. No. 456‐1034, Bio‐Rad, USA), and transferred to PVDF membrane (Mini Format, Cat. No. 1704156, Bio‐Rad, USA) using the BioRad Trans‐blot Turbo instrument. Membrane was blocked, and antibodies were diluted in EveryBlot Blocking Buffer (Cat. No. 12010020, BioRad, Japan), followed by incubation with either polyclonal rabbit anti‐TRIM25 (dilution 1:1000, NBP2‐20710, Novus Biologicals, CO, USA), or polyclonal rabbit anti‐beta‐actin (1:100,000, Cat. No. A300‐491A, Bethyl Laboratories, Montgomery, TX, USA) at 4°C overnight. Antibody binding was detected using a polyclonal goat anti‐rabbit IgG (1:10,000, Cat. No. 111‐035‐003, Jackson ImmunoResearch, West Grove, PA, USA) and with SuperSignal™ West Pico PLUS chemiluminescent substrate (Cat. No. 34580, Thermo Fisher Scientific), and scanned using C‐DiGit Blot Scanner (LI‐COR, Inc., NE, USA). Densitometry analysis of digitised images was performed using Fiji ImageJ 1.53 (64‐bit).

### 2.6 Statistical analyses

An association between TRIM25 expression and continuous parameters was analysed using the Kruskal–Wallis test. Cross‐tabulations with the Fisher–Freeman–Halton test were used to assess associations of *TRIM25* with MCPyV status, sex, sun exposure areas, p53 status, and metastasis at time of diagnosis. The missing data from metastasis information and p53 expression are presented in Table 1. The correlation between *TRIM25* gene expression and viral mRNA load or MCPyV DNA copy number was evaluated using Pearson correlation analysis. Overall and MCC‐specific survival were assessed with Kaplan–Meier analysis, with comparisons made by the log‐rank test. MCC‐specific and overall survival were calculated from the date of diagnosis to the time of disease‐specific death or any death, respectively, censoring the patients alive at the end of follow‐up. Cox proportional hazards model was used to assess univariate and multivariable survival. The Cox multivariate test was validated with an omnibus test. Statistical analyses were conducted with IBM SPSS statistics, version 28.0 (IBM Corporation, New York, NY, USA).

**TABLE 1:** Clinicopathological variables and their distribution in relation to *TRIM25* expression.

Factor	TRIM25 low	TRIM25 high	Total *N* = 102	*p*‐value
	*n* (%)	*n* (%)	*n*	
Sex				
Female	40 (51%)	38 (49%)	78	.641
Male	11 (46%)	13 (54%)	24	
Age				
Median (range)	80 (50–100)	79 (46–93)		.364
Sun exposure				
Yes	34 (53%)	30 (47%)	64	.413
No	17 (45%)	21 (55%)	38	
Tumour diameter (mm)				
Median (range)	16.5 (5–50)	13.0 (3–40)		.296
NA	11	4		
MCPyV status				
Negative	20 (74%)	7 (26%)	27	.004
Positive	31 (41%)	44 (59%)	75	
Metastases at diagnosis				
Yes	7 (64%)	4 (36%)	11	.299
No	39 (47%)	44 (53%)	83	
NA	5	3		
P53 status				
Positive	17 (61%)	11 (39%)	28	0.232
Negative	14 (45%)	17 (55%)	31	
NA	20	23		

## 3 RESULTS

### 3.1 Clinicopathological characteristics of the patient material

The study cohort consisted of 102 patients. Detailed clinicopathological variables and their association with *TRIM25* expression are summarised in Table 1. The only significant correlation with *TRIM25* was observed for MCPyV status (*p* = .004), with *TRIM25*‐positive tumours having a higher frequency of virus‐positive cases. Age, sex, metastasis status, sun exposure area, p53 status, and tumour size showed no significant correlations with *TRIM25* status.

### 3.2 Survival analysis

The median disease‐specific survival time for patients with *TRIM25* low‐expressing tumour was 1.88 years (range 4.68 months–5.52 years) and for patients with *TRIM25* high expression 2.82 years (range 4.08 months–14.81 years). Nineteen patients (37%) in the *TRIM25* low group died of MCC during follow‐up compared with 9 patients (18%) in the *TRIM25* high group. The median overall survival times for patients in *TRIM25* low and *TRIM25* high groups were 1.51 years (range 0.43 months–12.6 years, deaths 45 [88%]) and 3.48 years (range 1.12 months–27.25, deaths 43 [84%]), respectively.

**FIGURE 1:**
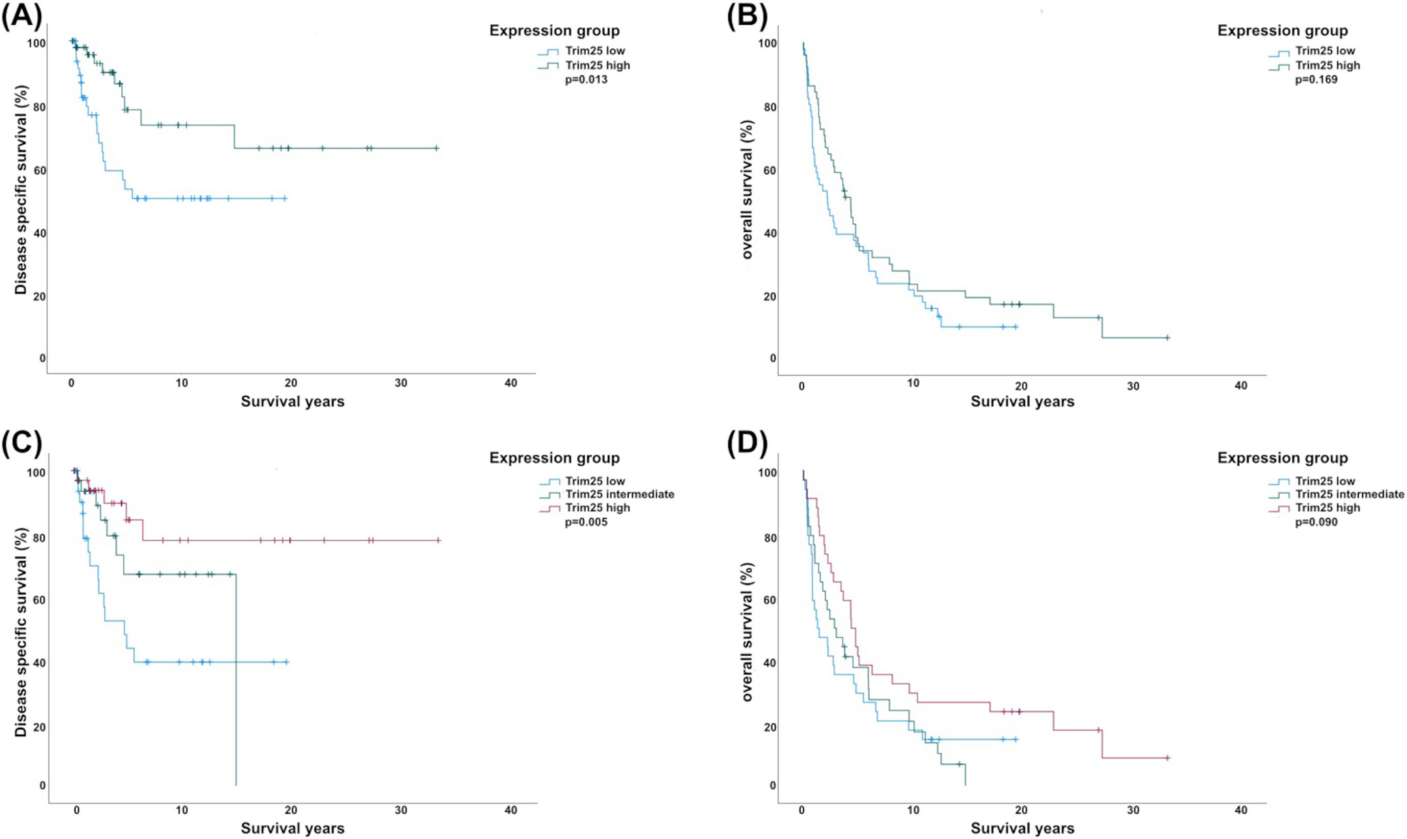
Kaplan–Meier curves of disease specific and overall survival in Merkel cell carcinoma (MCC) stratified by TRIM25 expression. Median expression division into low and high groups (A, B). (A) MCC‐specific survival where the TRIM25 high group has a favourable survival (5‐year survival: 78% vs. 53%; *p* = .013). (B) Overall survival, where there is no clear difference in survival between the groups (*p* = .169). Tertile division into low, intermediate and high TRIM25 expression groups (C, D). (C) MCC‐specific survival where the survival rate decreases stepwise from the TRIM25 high expressing group to low (5‐year survival: Low 44%; intermediate 67%; high 84%; *p* = .005). (D) Overall survival where there is no statistical difference in the survival rates between the groups (*p* = .090). The log rank test was used to calculate the *p*‐values.

*TRIM25* high expression tumours were associated with better disease‐specific survival than *TRIM25* low expression tumours (5‐year survival: 78% vs. 53%; *p* = .013; Figure 1A). There was no significant difference in overall survival between *TRIM25* low and high expression patients (5‐year survival: 35% vs. 38%; *p* = .169; Figure 1B). To evaluate the validity of the median used as a cut‐off for TRIM25 stratification, survival analyses were also performed with *TRIM25* expression stratified into tertiles. Despite using different cut‐offs for stratifying *TRIM25* expression, the survival outcomes remained consistent; the lowest *TRIM25* expression was associated with the worst disease‐specific outcome and the highest *TRIM25* expression with the best outcome (5‐year survival: low 44%; intermediate 67%; high 84%; *p* = .005; Figure 1C). Consistent with previous analyses, there were no significant differences between overall survival and *TRIM25* expression (Figure 1D).

Next, we investigated survival of patients with MCPyV‐positive or ‐negative tumours due to the strong association between *TRIM25* expression and viral status. Among patients with MCPyV‐negative tumours, those with *TRIM25* low tumour expression tended to have poorer disease‐specific survival than those with *TRIM25* high tumour expression (5‐year survival: 16% vs. 100%; *p* = .071; Supplementary Figure 1A). However, no significant differences in disease‐specific survival were observed among patients with MCPyV‐positive tumours or in overall survival, regardless of viral status (all *p*‐values > .05; Supplementary Figure 1).

The Cox proportional hazard model was used to investigate whether *TRIM25* expression is independently associated with disease‐specific survival. Male sex (hazard ratio [HR] = 3.86, 95% confidence interval [95% CI] = 1.83–8.16, *p* <.001), metastasis at diagnosis (HR = 9.84, 95% CI = 4.02–24.06; *p* < .001), and negative MCPyV status (HR = 4.41, 95% CI = 2.07–9.36, *p* <.001) were all associated with worse outcome and were entered with TRIM25 status into multivariable Cox regression analysis. Based on the analysis, metastasized tumours, male sex, and *TRIM25* low expression tumours were all independently associated with worse disease‐specific outcome (all *p*‐values <.05; Table 2), whereas negative viral status was not (*p* = .079).

**TABLE 2:** Multivariable Cox regression analysis of Merkel cell carcinoma‐specific survival.

Covariate	*B*(SE)	HR of death (95% CI)	*p*‐value
TRIM25 status			
Low versus high	1.080 (0.480)	2.944 (1.149–7.541)	.024
Metastases			
Present versus absent	2.057 (0.459)	7.824 (3.185–19.219)	<.001
Sex			
Male versus female	1.507 (0.438)	4.514 (1.915–10.639)	<.001
MCPyV positivity			
Yes versus no	−0.776 (0.441)	0.460 (0.194–1.093)	.079

### 3.3 DEGs in *TRIM25* low and *TRIM25* high groups

**FIGURE 2:**
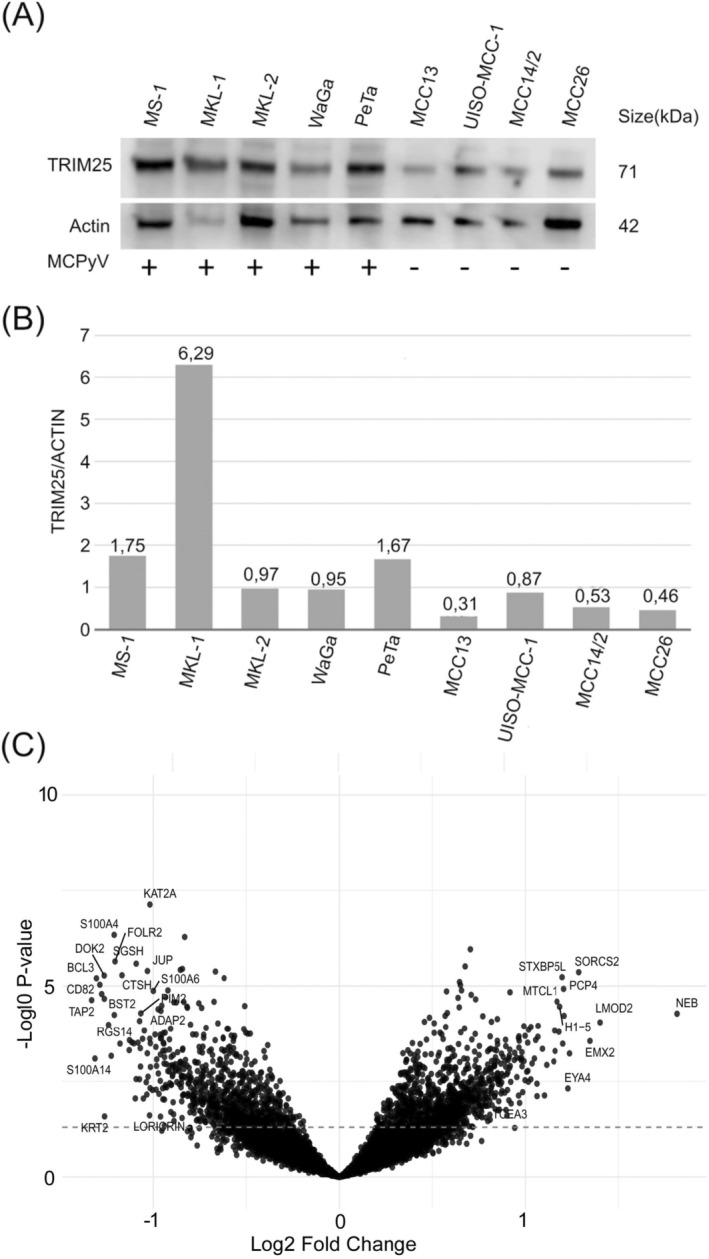
(A) Western blot of TRIM25 and actin in MCPyV‐positive and ‐negative MCC cell lines. (B) TRIM25/actin ratios quantified by densitometry; higher TRIM25 expression was observed in MCPyV‐positive lines (*p* = .014 [calculated with Kruskal–Wallis]). (C) Volcano plot of DEGs between *TRIM25*‐low and *TRIM25*‐high groups; red dashed line indicates significance threshold (*p* = .05). The TRIM25‐low group shows enrichment in immune response associated genes, while the TRIM25‐high group shows pathway enrichment in genes associated with cellular division and chromosomal maintenance.

DEGs associated with the *TRIM25* low group showed pathway enrichment to antigen‐presenting and other immune system function‐associated terms (Figure 2C). On the other hand, DEGs associated with the *TRIM25* high group contained many pathways associated with the regulation of mRNA processing, mitotic pathways, and chromosome maintenance. The top 10 most significantly enriched pathways in *TRIM25* low and *TRIM25* high groups are presented in Tables 3 and 4, respectively. The gene list of significant DEGs is presented in Supplementary Table 1. Finally, the correlation between TRIM25 mRNA expression and MCPyV mRNA load or relative MCPyV DNA copy number was assessed. The analysis revealed a weak but statistically significant positive correlation with MCPyV mRNA load (*r* = 0.264, *p* = .013), whereas no significant correlation was observed with MCPyV DNA copy number (*r* = 0.075, *p* = .457). These findings suggest a closer association of TRIM25 expression with viral transcriptional activity rather than with MCPyV genome copy number.

**TABLE 3:** Top 10 pathway terms significantly enriched in the TRIM25 low expression group.

Negative LogFC pathways	*p*‐value	Adjusted *p*‐value	Odds ratio	Combined score	Associated genes
Antigen processing: cross presentation	6.900e‐9	0.000001603	18.30	343.85	PDIA3, PSMD9, NCF4, UBC, TAP2, TAP1, CALR, B2M, TAPBP
Antigen processing and presentation	8.637e‐9	0.000001603	17.79	330.27	PDIA3, CD74, TAP2, TAP1, HLA‐DRA, CALR, B2M, HLA‐DRB1, TAPBP
Bystander B‐cell activation	0.001479	0.02892	45.31	295.26	HLA‐DRA, HLA‐DRB1
Eosinophils in the chemokine network of allergy	0.001479	0.02892	45.31	295.26	HLA‐DRA, HLA‐DRB1
PI3K subunit p85 role in regulation of actin organisation and cell migration	0.0002072	0.007814	31.57	267.81	ARPC3, ARPC1B, RHOA
Alpha‐4 beta‐7 integrin signalling	0.001892	0.03468	38.84	243.49	CD44, RHOA
Nef‐mediated downregulation of MHC class I complex cell surface expression	0.002354	0.04010	33.98	205.63	AP1S1, B2M
B‐lymphocyte cell surface molecules	0.002863	0.04379	30.20	176.86	HLA‐DRA, HLA‐DRB1
Salmonella infection	0.003419	0.04880	27.18	154.34	ARPC3, ARPC1B
BDNF signalling pathway	9.105e‐9	0.000001603	8.29	153.53	TMED9, IFITM2, IGFBP3, ARPC1B, TWF2, HSBP1, PTGS1, SLC7A5, SERPINH1, S100A4, TGFB1, CALR, JUNB, CLIC1

**TABLE 4:** Top 10 pathway terms significantly enriched in the TRIM25 high expression group.

Positive LogFC pathways	*p*‐value	Adjusted *p*‐value	Odds ratio	Combined score	Associated genes
Cell cycle	0.00001574	0.002977	4.59	50.77	MCM8, SMARCA5, SMC1A, NDC80, POLA1, CCNB1, CENPF, RBBP4, CLSPN, RBBP7, PCNT, BUB1, CDK5RAP2
Polo‐like kinase 1 (PLK1) pathway	0.00001588	0.002977	18.17	200.84	TPX2, CCNB1, CLSPN, BUB1, NDC80
Telomerase regulation	0.00009963	0.01245	12.01	110.62	JUN, RBBP4, NCL, NBN, RBBP7
Aurora B signalling	0.0001480	0.01387	16.91	149.12	CBX5, NCL, BUB1, NDC80
Myc active pathway	0.0003064	0.02298	9.30	75.21	CCND1, NCL, RCC1, NBN, PTMA
Capped intron‐containing pre‐mRNA processing	0.0003945	0.02466	6.79	53.25	SF3B2, TPR, CSTF1, UPF3B, HNRNPR, SMC1A
Messenger RNA processing	0.0005285	0.02831	5.36	40.46	SF3B2, TPR, CSTF1, HNRNPR, SMC1A, HRNRPAB
Chromosome maintenance	0.0007408	0.03472	7.58	54.65	POLA1, RBBP4, SMARCA5, RBBP7, SMC1A
Messenger RNA splicing: major pathway	0.001255	0.05114	9.23	61.69	CSTF1, URPF3B, HNRNPR, SMC1A
HIV life cycle	0.001364	0.05114	6.57	43.35	TAF13, TPR, RCC1, PSIP1, SSRP1

### 3.4 TRIM25 expression in cell lines

Finally, we assessed whether protein expression in MCC cell lines reflects our mRNA findings. MCPyV‐positive cell lines (MS‐1, MKL‐1, MKL‐2, WAGA, PeTa) exhibited significantly higher TRIM25 protein levels than the MCPyV‐negative cell lines (MCC13, MCC14/2, MCC26 and UISO‐MCC‐1), with a relative mean TRIM25/ACTIN ratio of 2.33 versus 0.54 (*p* = .014; Figure 2A,B).

## 4 DISCUSSION

Our findings indicate that TRIM25 may play a significant role in MCC tumourigenesis and serves as an independent prognostic factor for improved survival. Notably, low *TRIM25* expression correlates with poor prognosis, especially in MCPyV‐negative cases, suggesting a critical interplay between *TRIM25* expression levels and viral status in MCC pathogenesis. Moreover, gene expression profiles between *TRIM25* low and high expression cases are distinct, showing increased expression of genes associated with regulation of antigen‐presenting functions and mitotic activity, respectively. These findings highlight potential functional differences linked to TRIM25 expression that could influence tumour behaviour and patient outcomes.

Interestingly, we found a significant association between high TRIM25 expression and MCPyV positivity in both patient samples and MCC cell lines, which suggests a strong interplay between TRIM25 and viral status. Also, viral mRNA load in tumour tissue associated with *TRIM25* expression, whereas viral DNA copy number showed no such association. These findings suggest that TRIM25 expression is more closely linked to transcriptional activity rather than viral genome abundance. One possible explanation is the persistent expression of viral antigens in MCPyV‐positive tumours, which may activate antiviral signalling. TRIM25 is a key player of innate immunity in activation of the RIG‐I‐MAVS complex and further interferon production for antiviral functions.^9^, ^10^, ^11^, ^12^, ^13^ This process is regulated by Dot1L, which has been shown to slow viral replication when overexpressed.^28^ Similarly, in influenza virus and rabies virus‐infected cells, TRIM25 expression increases during infection,^28^, ^29^ and the presence of MCPyV may likewise increase *TRIM25* expression. It has also been speculated that TRIM25 could be upregulated by lncRNA XIST in hepatitis B virus (HBV)‐related hepatocellular carcinoma (HCC),^30^ although another study suggests that TRIM25 expression decreases in the peripheral blood mononuclear cells of HBV‐infected patients, and their serum further reduces TRIM25 expression in the HEPG2 cell line.^31^ However, contradictory findings in other cancers, such as reduced TRIM25 expression in HBV‐infected cells, suggest virus‐specific regulatory differences.

In our study, high *TRIM25* expression in MCC was associated with better disease‐specific outcome. The finding differs from other cancers, namely non‐small cell lung cancer and HCC, where high *TRIM25* expression has been shown to correlate with adverse outcome.^15^, ^21^ The poorer disease‐specific survival in MCPyV‐negative MCC cases, characterised by higher mutational burden and worse outcome, may partly explain this discrepancy.^5^, ^32^ Importantly, the Cox multivariate analysis confirmed that *TRIM25* is an independent prognostic factor for survival, even when viral status has been considered. Subgroup analysis revealed that low *TRIM25* expression was associated with poorer prognosis in MCPyV‐negative cases, while no such correlation was observed in MCPyV‐positive cases. However, small sample size and limited number of survival events in the subgroups necessitate validation in larger cohorts.

The *TRIM25* gene is at locus 17q22.^33^ Although no clear evidence links MCC to chromosomal changes in the long arm of chromosome 17, approximately 25% of MCC patients have losses in the short arm of chromosome 17, where the *TP53* gene is located.^34^ *TP53* mutations are characteristic of MCPyV‐negative MCC.^5^ Therefore, it would be interesting to investigate whether *TRIM25* gene losses or truncating mutations occur in MCC, potentially leading to reduced or absent gene expression. Crosstalk between TRIM25 and p53 could play a role in MCC tumourigenesis, as previous research suggests that TRIM25 can modulate p53‐dependent processes. For example, in the HTC116 colon carcinoma cell line, TRIM25 downregulation has been shown to induce p53‐dependent cell death.^35^ Further research is needed to explore whether TRIM25 has a disease‐specific duality in survival outcomes and whether it modulates p53 activity in MCC.

The role of TRIM25 in cancer appears to be highly context‐dependent, with malignancy‐specific functions. In HCC, which is often caused by hepatitis viruses, downregulation of TRIM25 leads to upregulation of the metastasis‐associated protein 1 (MTA‐1), whereas in glioblastoma, gastric cancer, and breast cancer cells, TRIM25 downregulation inhibits proliferation and migration in vitro.^33^, ^36^, ^37^ As both HCC and MCC are associated with viral oncogenesis, it is not far‐fetched to suggest that a similar viral‐related mechanism could influence bidirectional behaviour of TRIM25. This duality could explain discrepancies between our findings in MCC and earlier reported survival findings in other cancers. It is also tempting to hypothesise that TRIM25 may improve disease‐specific survival in MCPyV‐positive MCC, possibly due to its role in modulating antiviral immune responses and tumour biology.

Our transcriptomic analysis revealed that in the *TRIM25*‐low and predominantly MCPyV‐negative group, the expression of genes of the antigen‐presenting pathways was higher than in the *TRIM25*‐high and MCPyV‐positive group. This finding aligns with a previous study that showed downregulation of the MHC‐I pathway genes after transfection of the MCPyV‐negative MCC13 cell line with LT antigen.^38^, ^39^ Conversely, in the *TRIM25*‐high group, genes associated with cell cycle regulation were upregulated. This finding could be explained by the fact that high *TRIM25* expression is associated with the presence of MCPyV, and the viral LT antigen is known to promote cell cycle progression through retinoblastoma protein inactivation.^40^ As this study was conducted with bulk RNA sequencing, the origin of gene expression signals within the tumour microenvironment remains unknown. Single‐cell data analysis could provide clarity on this issue. In addition, it remains unclear whether TRIM25 has a direct role in regulation of these pathways or if it interacts with MCPyV to influence these signalling pathways. Further detailed studies are needed to address these questions and to better understand the underlying molecular mechanisms.

The evolving therapeutic landscape of MCC, particularly the introduction of anti‐PD‐1 and anti‐PD‐L1 immunotherapies, underscores the need for reliable predictive biomarkers. In our cohort, patients did not receive immune checkpoint inhibitors (ICIs), limiting direct evaluation of association between TRIM25 expression and response to immunotherapy. Prior to 2016, when ICIs were introduced in the treatment of MCC, the main treatment for metastatic disease, in addition to excision and radiotherapy, was platinum‐based chemotherapeutic agents.^41^ High PD‐L1 expression in tumour tissue is currently used in clinical practice to guide patient selection for immunotherapy. However, PD‐L1 alone is rarely a sufficient biomarker and other biomarkers such as tumour mutation burden (TMB), tumour‐infiltrating lymphocytes (TILs), and gene expression profiles are integrated to refine patient stratification for immunotherapy.^42^

Although our cohort did not include patients treated with ICIs, the observed association between *TRIM25* expression and MCPyV status, as well as disease‐specific survival, provides a compelling rationale for further investigation in ICI‐treated populations. MCPyV‐positive MCCs are known to have lower TMB but higher TIL counts, both of which may influence ICI responsiveness.^5^, ^6^, ^42^ TRIM25, as a regulator of antiviral signalling and interferon production, may contribute to a more immunogenic tumour microenvironment, particularly in virus‐positive cases. This could enhance antigen presentation and immune recognition, potentially improving responsiveness to PD‐1/PD‐L1 blockade. Conversely, in MCPyV‐negative tumours, which are characterised by higher TMB and poorer prognosis, low TRIM25 expression may reflect impaired antiviral signalling and reduced immune activation. TRIM25 may also directly influence PD‐L1 signalling as it has been shown to promote PD‐L1 mRNA degradation in gastric cancer and facilitate immune suppression via the TRIM25‐NF‐kB‐PD‐L1 pathway in gliomas.^43^, ^44^

A methodological limitation of our study lies in the nf‐core/viralintegration pipeline used to quantify MCPyV transcriptional activity. Although it effectively detects viral–host chimeric transcripts indicating integrated virus, the method relies on short‐read RNA sequencing, which is sensitive to sequencing depth, read length, and reference completeness. MCPyV integration frequently, though not always, occurs near or within the LT gene region, generating truncations and rearrangements that produce truncated LT, a key viral oncogene in MCC.^45^, ^46^ Therefore, detected chimeric transcripts are likely enriched for T‐antigen sequences, even though integrations can involve other viral regions. At the same time, complex integration events and short‐read constraints limit accurate assignment of reads to specific viral genes. Short reads also fail to span concatemerized viral genomes, leading to underestimation of transcripts arising from repeated viral sequences.^46^ Consequently, targeted assays such as qPCR remain necessary for precise viral gene‐level quantification.

Together, these findings indicate that TRIM25 is a promising positive prognostic biomarker in MCC. Future studies should focus on clarifying its functional role in MCC tumourigenesis. Additionally, exploring TRIM25's association with responses to anti‐PD‐1/PD‐L1 immunotherapy could help to develop diagnostic tools and potentially enable identification of the patients most likely to benefit from therapy.

## AUTHOR CONTRIBUTIONS

**Klaus W. Fagerstedt:** Conceptualization; investigation; writing – original draft; methodology; writing – review and editing; visualization; formal analysis; software. **Sami Kilpinen:** Software; data curation; writing – review and editing; formal analysis. **Johanna Arola:** Funding acquisition; writing – review and editing. **Benjamin Z. Sundqvist:** Investigation; writing – review and editing. **Tom Böhling:** Funding acquisition; conceptualization; writing – review and editing. **Leif C. Andersson:** Conceptualization; funding acquisition; writing – review and editing. **Harri Sihto:** Conceptualization; methodology; supervision; writing – review and editing; funding acquisition.

## FUNDING INFORMATION

Funding provided by the Cancer Foundation Finland (4709194), Medicinska Understödsföreningen *Liv och Hälsa* rf (4708936), Finska Läkaresällskapet (4707201 TB, 4709747), and the Sigrid Juselius Foundation (4709424, 4706829) is gratefully acknowledged.

## CONFLICT OF INTEREST STATEMENT

The authors declare no conflicts of interest.

## ETHICS STATEMENT

The study protocol was approved by the Ethics Committee of Helsinki University Central Hospital and the local review board (HUS 296/E6/2001 and HUS/221/2017). The need for informed consent was waived by the Ministry of Health and Social Affairs, who granted permission to collect patient data (STM/398/2005), and the National Authority for Medicolegal Affairs, who granted the permission to collect and analyse tissue samples (3320/32/300/03 and 4942/05.01.00.06/2009).

## Supporting information


**Supplementary Figure 1.** Kaplan–Meier curves of disease specific and overall survival in Merkel.

**Supplementary Table S1:** The gene list of significant DEGs regarding TRIM25 expression.

## Data Availability

Publicly available datasets were analysed in this study. These data can be found at PRJNA775071. Further information is available from the corresponding author upon request.
