# Supplementary material for: Antiviral regulator TRIM25 as a prognostic marker of better survival in Merkel cell carcinoma: Association with MCPyV status
Source: Int J Cancer. 2026 Feb 14;158(12):3268–78. doi: 10.1002/ijc.70384 (PMC13106916; doi:10.1002/ijc.70384)
Supplement: Supplementary file 1 — Supplementary Figure 1. Kaplan–Meier curves of disease specific and overall survival in Merkel. [file IJC-158-3268-s001.pdf]

Antiviral regulator TRIM25 as a prognostic marker of better survival in Merkel cell carcinoma: Association with MCPyV status

Klaus W Fagerstedt , Sami Kilpinen , Johanna Arola, Benjamin Z Sundqvist, Tom Böhling, Leif C Andersson, Harri Sihto.

Table of content:

1. Supplementary figure 1
2. Supplementary table 1 (available in separate file)

1.

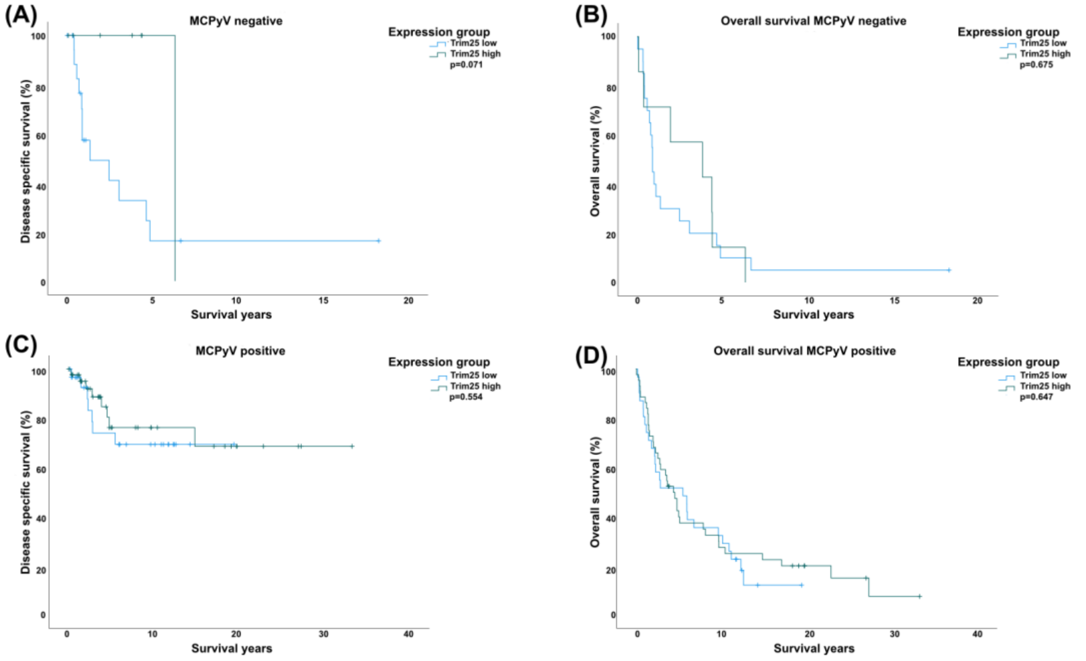

Supplementary Figure 1. Kaplan-Meier curves of disease specific and overall survival in Merkel cell carcinoma (MCC) stratified by TRIM25 expression and divided into MCPyV negative and positive groups. TRIM25 expression-median expression division is used in all curves. (A) MCCspecific survival in MCPyV-negative group where the survival looks like to be profitable for the TRIM25-high group, but no statistical significance is not seen (p=0.071). (B) Overall survival for the MCPyV-negative group shows no difference in survival between TRIm25 expression groups (p=0.675). (C) MCC-specific survival for the MCPyV-positive group shows no statistical difference in survival between TRIm25 expression groups (p=0.554). (D) Overall survival for the MCPyV-positive groups is indifferent in statistically significant survival between TRIm25 expression groups(p=0.647). The p-values are calculated using log rank test.
